# Hyalinizing pancreatic adenocarcinoma in a cat

**DOI:** 10.1177/20551169251325333

**Published:** 2025-04-16

**Authors:** Taylor C Chan, Elisa Heacock, Ashleigh Cournoyer, Koranda Walsh, Amy C Durham

**Affiliations:** 1 Department of Pathobiology, University of Pennsylvania School of Veterinary Medicine, Philadelphia, PA, USA; 2 Department of Clinical Sciences and Advanced Medicine, University of Pennsylvania School of Veterinary Medicine, Philadelphia, PA, USA; 3 Upstate Veterinary Specialties, Latham, NY, USA; 4 Department of Diagnostic Medicine & Pathobiology, Rowan University Shreiber School of Veterinary Medicine, Glassboro, NJ, USA

**Keywords:** Pancreatic adenocarcinoma, exocrine pancreas, hyalinizing, histopathology, cytology, diagnostic imaging

## Abstract

**Case summary:**

A 6-year-old female spayed domestic shorthair cat was presented for abdominal distension and weight loss. Abdominal radiographs and ultrasound revealed two cranial abdominal masses and another mass adjacent to the jejunum. Cytologic features of the cranial abdominal masses were consistent with exocrine pancreatic tissue. Four months later, a repeat abdominal ultrasound revealed progressive enlargement of the abdominal masses and medial iliac lymphadenopathy. On exploratory laparotomy, two abdominal masses were associated with the pancreas and incorporated large blood vessels supplying the liver, pancreas and spleen. The masses were non-resectable and incisional biopsies were obtained. The histologic features were diagnostic for a hyalinizing subtype of exocrine pancreatic adenocarcinoma. Chemotherapy was not pursued. Over 28 months after the initial detection of abdominal masses, the cat was still alive and reportedly doing well.

**Relevance and novel information:**

To the authors’ knowledge, this is the first report of a hyalinizing subtype of pancreatic adenocarcinoma in a cat. This subtype is considered to behave less aggressively in dogs, and this case may support that a similar, more indolent behavior may be seen in cats.

## Case description

**Figure 1:**
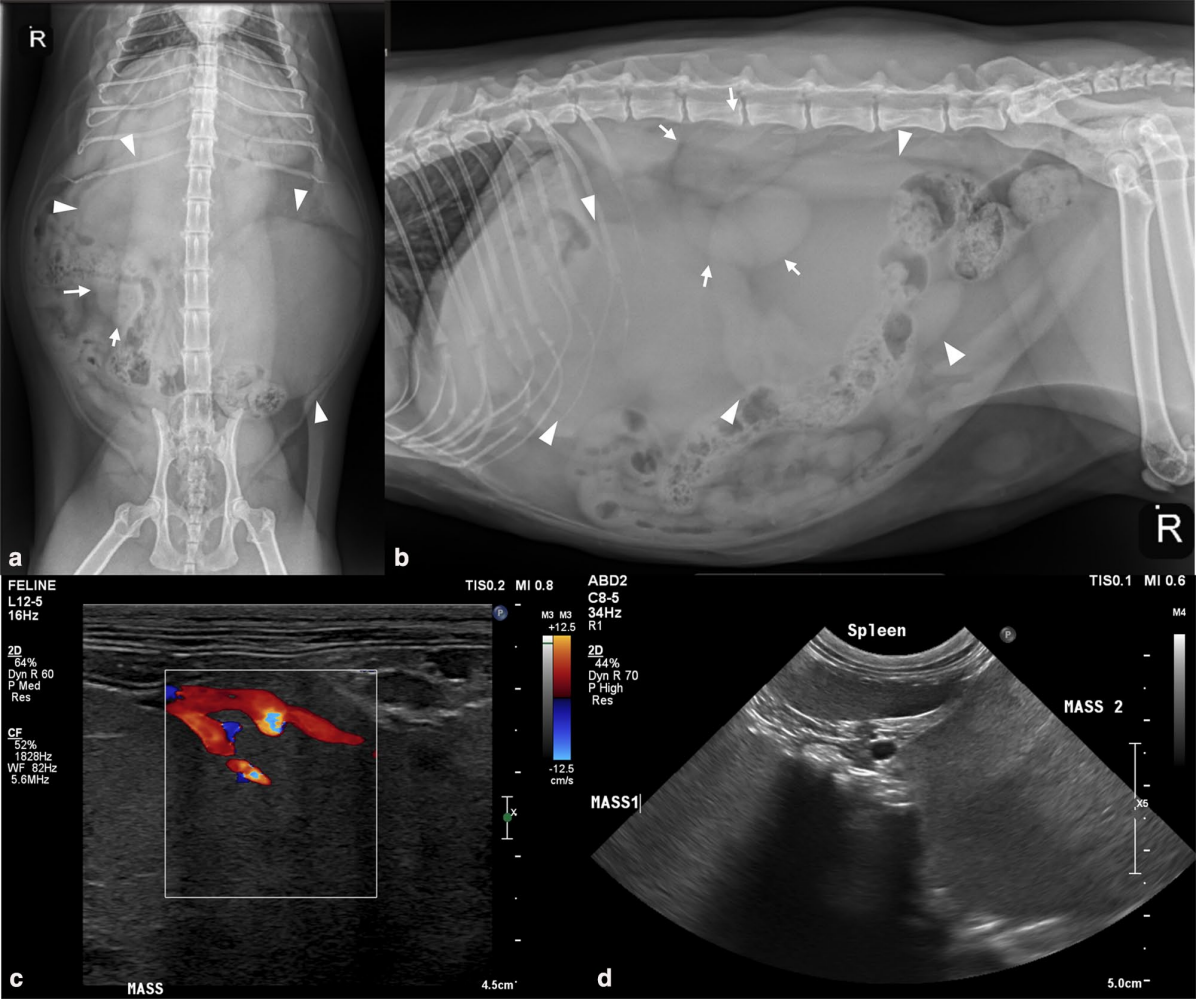
(a) Ventrodorsal and (b) right lateral radiographic projections of the abdomen showing two large soft tissue opaque intraabdominal masses (arrowheads) causing significant intestinal displacement. Kidneys are demarcated (arrows). (c,d) Ultrasound images of the presumed mass hilus and demonstrating the relationship between the masses and the spleen (obtained transcutaneously from the left side of the patient)

A 6-year-old female spayed domestic shorthair cat was presented to the primary veterinarian for a distended abdomen and mild weight loss over several months. A complete blood count (CBC), chemistry and thyroid panel were performed and demonstrated elevated lipase (318 U/l; reference interval [RI] 0–45). The cat was re-evaluated 1 month later by the primary veterinarian and two firm, mid-abdominal masses were palpated. Abdominal radiographs performed by the primary veterinarian revealed two large lobular soft tissue masses filling the abdomen, centered on the right cranial and mid-left abdomen (Figure 1a). The masses caused leftward and ventral displacement of the small intestines and colon (Figure 1b). An ovoid soft tissue opacity within the inguinal region was presumed to be an enlarged lymph node. These findings were interpreted as presumed lymphadenopathy of the jejunal and inguinal lymph nodes, and differential diagnoses of lymphoma, other round cell neoplasia or systemic infectious disease processes, such as granulomatous disease (eg, fungal infection) and feline infectious peritonitis, were considered. The patient was otherwise clinically normal and referred to a specialty oncology service.

Six weeks after the initial presentation, the cat was presented to the oncology service at the Matthew J Ryan Veterinary Hospital with a markedly distended abdomen, two large, firm, palpable masses in the mid-cranial abdomen and mild bilateral inguinal lymphadenopathy. Bloodwork showed mild neutrophilia (11.84 K/µl, RI 2.30–11.60) and minimal pre-renal azotemia with slightly elevated creatinine and normal blood urea nitrogen (BUN) (creatinine 2.1 mg/dl, RI 1.0–2.0; BUN 19 mg/dl, RI 15–32; urine specific gravity [USG] 1.039). Abdominal ultrasound revealed two large hyperechoic, round, lobular masses of undetermined origin displacing numerous abdominal structures and a third similar smaller structure within the mesentery adjacent to the jejunum. The masses had large mesenteric vessels tracing into the center, creating a hilus-like internal architecture and were each surrounded by fat, suspected to represent lymph nodes (Figure 1c,d). Infectious granulomatous disease was prioritized over neoplasia owing to the hyperechoic appearance of these masses. Additional findings included scant peritoneal effusion, mild splenomegaly and changes suggestive of chronic enteropathy, non-specific hepatopathy and mild chronic renal disease.

**Figure 2:**
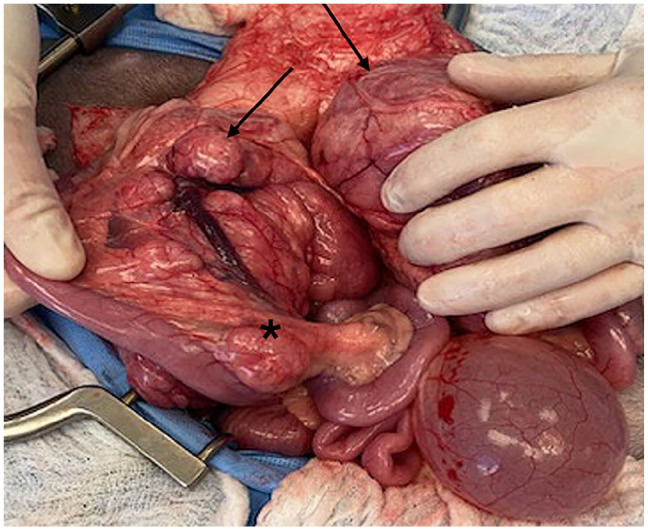
(a) On cytology, low to moderate numbers of aggregated epithelial cells with mild atypia on a background of abundant thick, mucinous material (WG, × 200); (b) neoplastic exocrine pancreatic epithelial cells forming acini separated by lightly eosinophilic extracellular material. Atypia is mild to moderate (H&E, × 200); (c) the extracellular material is not congophilic (Congo red, × 200). H&E = hematoxylin and eosin; WG = Wright Giemsa

Ultrasound-guided aspirates of the abdominal masses were performed. Cytologically, there were low to moderate numbers of aggregated epithelial cells, occasionally in pseudoacinar arrangement, on a background of abundant, thick mucinous material (Figure 2a). Epithelial cells exhibited mild atypia with a moderate amount of amphophilic, vacuolar to granular cytoplasm and centrally located round-to-oval nuclei containing stippled to lacy chromatin and a small blue nucleolus, suggesting a proliferation of exocrine pancreatic tissue. Cytologic evaluation of an enlarged inguinal lymph node indicated chronic inflammation. At the time, exploratory surgery and mass biopsies were declined.

**Figure 3:**
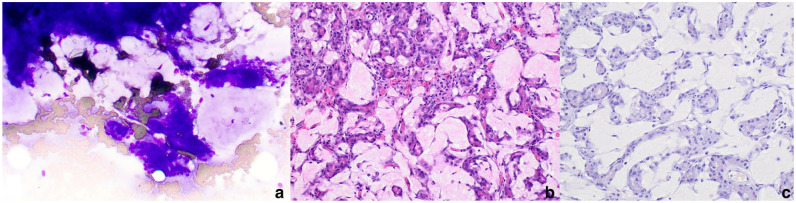
Two cranial abdominal masses (black arrows), measuring 10 × 10 × 4 cm, incorporate the pancreas and hepatic, pancreatic and splenic blood supplies. A mesenteric lymph node along the jejunum is markedly enlarged (asterisk)

The cat was re-evaluated by the oncologist 5 months after initial presentation and was clinically normal other than intermittent soft stools. Bloodwork and physical examination were unchanged from the prior visit. A repeat ultrasound showed mildly progressive enlargement and heterogeneity of the masses, and a subsequent exploratory laparotomy was performed. The cranial abdomen had two masses measuring 10 × 10 × 4 cm in size incorporating the pancreas and hepatic, pancreatic and splenic blood supplies. The third smaller mass appeared to be an enlarged jejunal lymph node (Figure 3). Other findings included diffusely thickened small intestines and slightly rounded liver lobes with an enhanced reticular pattern. The cranial abdominal masses were non-resectable because of incorporation of multiple critical blood vessels to adjacent visceral organs. Multiple guillotine and punch biopsies were obtained from both abdominal masses, the jejunum and the jejunal lymph node.

A histologic examination of the biopsy samples from the abdominal masses consisted of neoplastic exocrine pancreatic tissue with no evidence of normal pancreatic or nodal tissue. The neoplasm comprised polygonal cells arranged in acini separated by a lightly eosinophilic, fibrillar to homogenous extracellular matrix (Figure 2b). Neoplastic cells had moderate to abundant cytoplasm containing eosinophilic zymogen granules and oval nuclei with variably prominent nuclei. Anisocytosis and anisokaryosis were moderate, with a mitotic count of 1 per 2.37 mm^2^ (10 FN22/40× fields). Numerous foamy macrophages and few lymphocytes, plasma cells and eosinophils were scattered throughout the neoplasm. The extracellular matrix was negative for Congo red staining, with a few thin wisps of trichrome-positive collagen noted, consistent with non-amyloid hyaline material (Figure 2c). These features were consistent with a hyalinizing pancreatic adenocarcinoma. Neoplastic cells were not observed in the jejunal lymph node, and a diagnosis of drainage reaction was made based on histologic evaluation and PCR for antigen receptor rearrangement (PARR). Changes in the jejunum were consistent with eosinophilic inflammatory bowel disease.

At the oncology consultation 2 weeks after the exploratory laparotomy/biopsy, the patient was clinically normal. Chemotherapy (toceranib phosphate) was discussed but ultimately not pursued. At 28 months after the initial presentation, the owner reported the cat was normal at home and had not received treatment for the pancreatic adenocarcinoma.

## Discussion

The hyalinizing subtype of pancreatic adenocarcinoma is rarely diagnosed in dogs; however, to date, it has not been reported in cats. Exocrine pancreatic neoplasia is rare in cats, with estimates of 12.6/100,000 cats per year at risk and has a high metastatic rate and poor prognosis.^1,2^ Common sites for metastasis include the peritoneum (carcinomatosis), liver, lungs and regional lymph nodes.^3^ Because of the combination of significant systemic disease, peritoneal effusion, pancreatitis and metastatic disease at presentation, humane euthanasia is commonly elected without surgical or medical intervention, further perpetuating poor outcomes.^2^

In a case series of nine cats with localized exocrine pancreatic adenocarcinoma and no metastatic disease on presentation, survival times were in the range of 25–964 days, with a median survival time (MST) of 316.5 days after surgical removal with macroscopically disease-free marginal excisions.^2^ In another retrospective study, 34 cats diagnosed with exocrine pancreatic adenocarcinoma had an overall MST of 97 days, with cats that underwent chemotherapy or surgical removal reaching up to 165 days. Of the cats, 32% had metastatic disease and three cats survived longer than 1 year.^3^

Histologic subtypes reported in cats include acinar, tubular, mixed and cystic differentiation.^4^ Despite the variability in clinical presentations and histologic subtypes, the degree of neoplastic cell differentiation and mitotic rate were not statistically significant in relation to disease progression or survival.^3^ In a comparison of cystic and solid pancreatic neoplasms in cats, tumors were classified as adenomas or adenocarcinomas based on vessel infiltration and metastasis.^4^ Gross size, cellular pleomorphism and mitotic count were similar between benign and malignant tumors and were therefore not considered criteria for malignancy classification.^4^

The hyalinizing subtype of pancreatic adenocarcinoma is described in a single case series of six dogs.^5^ Key characteristics include tubules and acini of exocrine pancreatic epithelial cells, along with a tumor stroma composed of abundant hyaline material that is negative for immunohistochemical markers for amyloid.^5^ The stromal material stains faintly blue with Masson’s trichrome and is neither congophilic nor birefringent.^5^ Post-diagnosis survival times ranged from a few days to 16 months without treatment, and over 19 months after partial pancreatectomy. Metastasis was observed in 1/6 dogs that survived for 16 months after diagnosis.^5^ Mortality and/or euthanasia were associated with concurrent comorbidities, pancreatitis and postoperative complications rather than metastatic disease.^5^ In contrast, the survival time in dogs with conventional exocrine pancreatic adenocarcinomas ranged from 1 day to 19 weeks.^6^ The results from this small case series suggest that the hyalinizing subtype may be biologically less aggressive than conventional exocrine pancreatic adenocarcinomas.

The extended post-diagnosis survival seen in dogs with the hyalinizing subtype was also seen in this cat, which is still alive more than 28 months after initial presentation. We suggest that, as in dogs, hyalinizing pancreatic adenocarcinoma in cats may have a better prognosis than other subtypes. The absence of pancreatitis or significant comorbidities likely contributed to the cat’s prolonged survival. Given the significant systemic effects of pancreatitis, we postulate that tissue damage associated with exocrine pancreatic neoplasms, resulting in the release of pancreatic enzymes, may be a major factor in morbidity and, ultimately, the decision for humane euthanasia, regardless of histologic subtype or species.

Diagnosing feline exocrine pancreatic adenocarcinoma can be challenging owing to its non-specific clinical findings, which include anorexia, vomiting, jaundice, abdominal pain, serum biochemical disturbances and elevated white blood cell counts.^3^ In addition, diagnostic imaging is complicated by the location of the pancreas in the cranial abdomen, poor serosal detail in cases of concurrent pancreatitis or peritonitis, and the presence of peritoneal effusion.^3,7^ Abdominal masses can be visualized with radiographs, but interpreting these masses as pancreatic in origin vs mesenteric lymph nodes can be difficult.^7^ Our case report highlights several features that contributed to its diagnostic challenge. Extensive vascularization of the individual tumors was observed on ultrasonographic imaging and during surgery, creating a ‘hilus-like’ appearance resembling a lymph node. Clinically, mild neutrophilia, elevated lipase, pre-renal azotemia, weight loss and soft stool were non-specific findings, and the glabrous alopecia typically associated with feline pancreatic adenocarcinomas was not present.^8,9^ Observing neoplastic exocrine pancreatic epithelial cells on both cytologic and histopathologic examinations, rather than a lymphocytic proliferation or primary inflammation, rules out lymphoma and an infectious etiology, exemplifying the importance of microscopic examination in this case. Regardless of the diagnosis, the size of the masses and extensive involvement with abdominal vasculature were concerning for an aggressive disease process.

There is limited literature investigating the use of toceranib phosphate (Palladia; Zoetis) to treat cats with pancreatic adenocarcinoma. In a case series of 26 cats treated with toceranib phosphate for exocrine pancreatic adenocarcinoma, the overall MST was 97 days, with 45% of treated cats with gross disease experiencing stable disease or complete response and 55% of cats experiencing progressive disease.^10^ Toceranib phosphate was well-tolerated, with most adverse events reported as either grade 1 or grade 2 according to Veterinary Cooperative Oncology Group criteria.^10,11^ In a case report of a cat receiving toceranib phosphate for a non-resectable pancreatic mass, progressive disease was not detected until 11 months after initiating treatment.^12^ Ultimately, reasons for forgoing chemotherapy in this case included insufficient evidence supporting the efficacy of treatment, risk of side effects, difficulty medicating the patient and absence of overt clinical disease at the time of discussion.

## Conclusions

This is the first case report of a hyalinizing pancreatic adenocarcinoma in a cat and highlights the diagnostic challenges and unexpected outcome of the patient. Despite the diagnosis of exocrine pancreatic adenocarcinoma, the patient was alive with stable disease more than 28 months after the initial detection of abdominal masses, without chemotherapy or surgical resection, and no reported progression of clinical signs. Hyalinizing pancreatic adenocarcinomas are rare but may exhibit less aggressive biological behavior than other subtypes of exocrine pancreatic neoplasms, even in cats.
